# Linking crop traits to transcriptome differences in a progeny population of tetraploid potato

**DOI:** 10.1186/s12870-020-2305-x

**Published:** 2020-03-18

**Authors:** Erik Alexandersson, Sandeep Kushwaha, Aastha Subedi, Deborah Weighill, Sharlee Climer, Daniel Jacobson, Erik Andreasson

**Affiliations:** 1 Department of Plant Protection Biology, Swedish University of Agricultural Sciences, Sundsvägen 10, Alnarp, Sweden; 2 Present address: Department of Biostatistics, Harvard T.H. Chan School of Public Health, Harvard University, Boston, MA USA; 3 Department of Plant Breeding, Swedish University of Agricultural Sciences, Alnarp, Uppsala, Sweden; 4 National Institute of Animal Biotechnology, Hyderabad, India; 5 Biosciences Division, Oak Ridge National Laboratory, Oak Ridge, TN USA; 6 The Bredesen Center for Interdisciplinary Research and Graduate Education, University of Tennessee, Knoxville, Knoxville, TN USA; 7 University of Missouri-St. Louis, St. Louis, MO USA

## Abstract

**Background:**

Potato is the third most consumed crop in the world. Breeding for traits such as yield, product quality and pathogen resistance are main priorities. Identifying molecular signatures of these and other important traits is important in future breeding efforts. In this study, a progeny population from a cross between a breeding line, SW93–1015, and a cultivar, Désirée, was studied by trait analysis and RNA-seq in order to develop understanding of segregating traits at the molecular level and identify transcripts with expressional correlation to these traits. Transcript markers with predictive value for field performance applicable under controlled environments would be of great value for plant breeding.

**Results:**

A total of 34 progeny lines from SW93–1015 and Désirée were phenotyped for 17 different traits in a field in Nordic climate conditions and controlled climate settings. A master transcriptome was constructed with all 34 progeny lines and the parents through a de novo assembly of RNA-seq reads. Gene expression data obtained in a controlled environment from the 34 lines was correlated to traits by different similarity indices, including Pearson and Spearman, as well as DUO, which calculates the co-occurrence between high and low values for gene expression and trait. Our study linked transcripts to traits such as yield, growth rate, high laying tubers, late and tuber blight, tuber greening and early flowering. We found several transcripts associated to late blight resistance and transcripts encoding receptors were associated to *Dickeya solani* susceptibility. Transcript levels of a UBX-domain protein was negatively associated to yield and a GLABRA2 expression modulator was negatively associated to growth rate.

**Conclusion:**

In our study, we identify 100’s of transcripts, putatively linked based on expression with 17 traits of potato, representing both well-known and novel associations. This approach can be used to link the transcriptome to traits. We explore the possibility of associating the level of transcript expression from controlled, optimal environments to traits in a progeny population with different methods introducing the application of DUO for the first time on transcriptome data. We verify the expression pattern for five of the putative transcript markers in another progeny population.

## Background

Potato is the world’s third largest food crop. It has a high yield potential and constitutes an almost irreplaceable part of many countries’ cuisines [1]. Since the cultivated tetraploid potato in Europe originates from the limited germplasm at the time of introduction, it has a narrow genetic base [2]. Due to its high degree of heterogeneity and inbreed depression, DNA markers have so far had rather limited use in potato breeding [3]. Next-generation sequencing (NGS) offers new possibilities to do high throughput transcriptome profiling of plant progeny populations and offers new ways to also study transcript expression associated to phenotypic traits.

Linking transcripts to phenotypes can be a way to develop new transcript markers and transcript profiles, so-called molecular signatures, associated to a certain trait. This has been shown in other crops where, e.g., transcripts important in leaf development were identified in a maize mapping population [3, 4]. Recently, a number of transcript and metabolite markers associated to the complex trait drought tolerance in potato was described [5]. These putative transcript markers also provide the first step in revealing genes involved in complex traits of polygenic origin, which could possibly overcome part of the challenge in analysing polyploid species.

Many studies have found clearer expression differences between parents than between the progeny lines. Indeed, it has been reported that the majority of differentially-expressed genes between two parents are expressed at inter-parent levels in the hybrid offspring [6]. In maize, six different genotypes in the F_1_ generation revealed a correlation between genetic diversity and transcriptional variation, however, one-quarter of the genes showed non-additive effects in expression [4, 7, 8]. The extent of variation in gene expression profiles between parents and progeny and in between progeny may depend on the species, parental genotypes, environmental discrepancies and plant tissue studied.

Here, we explore the differences in transcriptome composition between members of a population from two tetraploid parents by generation of RNA-seq from controlled conditions. We do this by applying three different correlation methods, Pearson and Spearman correlation, and the novel DUO similarity metric to compare the transcripts associated to certain phenotypic traits. DUO classifies the values of a matrix as high, low or neutral, and then calculates the co-occurrence of high, low or high/low values. (Climer et al., submitted). The DUO metric is an adaptation of an existing and published metric called the Custom Correlation Coefficient – CCC [9]. The CCC is a Single Nucleotide Polymorphism (SNP) correlation metric designed specifically for the case where the data vector’s elements are divided into two main categories. The structure of the DUO formula is identical to that of the CCC formula, except that in this case, the categories are high/low expression level/phenotype level instead of allele. This is a useful metric to use in addition to Pearson and Spearman correlation metrics as it naturally deals with categorical data.

In this way, we find potential new transcript markers and profiles associated with different traits in a progeny population of potato that at the same time indicate the underlying molecular mechanisms with the specific traits. Ideally, transcript markers should be robust enough to be useful in a controlled setting to further enhance the screening process as several generations of plants per year can be generated in controlled conditions. Thus, whereas the transcriptome was captured from unstressed plants grown in controlled conditions, the phenotypic data depending on trait were recorded either from field or controlled climate settings.

Late blight resistance (caused by *Phytophthora infestans*) has previously been studied in this population, and the receptor resistance (R-) gene behind the resistance has been cloned [10]. From a previous quantitative proteomics study of this population based on selective reaction monitoring (SRM), we concluded that even in the absence of the pathogen, the resistance gene was affecting the abundance of a number of selected secreted proteins [11]. Here, we found transcriptome effects of this R-gene again without any pathogens present. In addition, we report phenotypic data from a resistance screening in controlled conditions of *Alternaria solani* inoculation in leaves and tubers as well as *Dickeya solani* inoculation. In field trials we recorded agronomical important traits for potato and analysed associated transcripts for height, growth rate, flowering time, leaf texture, leaf lesions, number of tubers, number of green tubers and yield.

## Results and discussion

### Phenotyping correlations in the progeny population

We phenotyped 34 lines for 17 plant traits in three trait categories (Table 1), namely ***Biotic Stress:****P. infestans* resistance in leaves (PIR), tuber blight caused by *P. infestans* (TBS), Dickeya response (DR, suseptibility), Lesions after Alternaria infection in leaves (LAI), Alternaria infection volume (AIV) in tubers, degree of HR-like lesions (HRL); ***Tuber***: Number of tubers (NoT), Tuber greening in field (TGF), Visible tubers close to soil surface (VT), Yield per plant 2013 (YP13), Yield per plant 2014 (YP14); ***Leaf/Shoot/Flower:*** Senescence level in the late season (SLS), Growth rate (GR), Height (H), Level of necrotic leaves in the late season (NLL), Leaf texture (LT), Flowering time (FT). Among the 17 traits, three were categorical and the rest were quantitative (Table 1). The progeny lines show clear difference in scoring values for most traits and scoring values of the parents are often different from the average values of the traits in the segregating progeny lines (Table 1). The phenotype data for all traits underlying the analysis is included as Additional file 1: Table S1.

**Table 1:** Mean, maximum and minimum of the traits determined form the 34 progeny lines and two parents

Abbreviation	Trait	Unit	Condition	Mean ± SD	Max	Min	Désirée	SW93–1015	N (plants/ tubers per line)	Time of observation
DR	Dickeya resistance	score	Lab	1.9 ± 1.7	6.8	0.00	5.4	0.7	12	NA
LAI	Lesions after Alternaria infection (leaf)	%	Greenhouse	3.3 ± 0.8	4.9	1.9	1.9	3.6	10	NA
AIV	Alternaria infection volume (tuber)	mm3	Lab	535 ± 371	1946	112	322	523	6	NA
TBS	Tuber blight score	score	Lab	2.5 ± 0.90	5.0	0.3	2.2	0.3	7	NA
VT	Visible tubers/plant	number	Field	3.2 ± 2.4	14.5	0.3	0.3	2.8	10	2 Sep 2013
TGF	Tuber greening/plant	number	Field	0.08 ± 0.12	0.60	0	0	0.10	10	2014
YP13	Yield/plant 2013	kg	Field	1.46 ± 0.7	4.1	0.5	1.3	1.0	10	2013
YP14	Yield/plant 2014	kg	Field	1.21 ± 0.5	2.1	0.33	1.2	0.3	10	2014
NoT	Number of tubers	number	Field	112 ± 52	245	29	96	104	10	2014
H	Height	cm	Field	43.5 ± 11.2	61.5	18.5	46.6	54.1	10	22 July 2014
GR	Growth rate	cm/day	Field	0.75 ± 0.31	1.23	0.15	0.71	1.14	10	2014
HRL	Degree of HR-like lesion	Score	Field	4.0 ± 1.5	6.0	1.0	3.0	2.0	10	22 Jul 2014
SLS	Senescence late season	%	Field	38.5 ± 28.2	100.0	5.0	30.0	8.8	10	26 Aug 2014
NLL	Necrotic leaves, late-season	%	Field	40.5 ± 39.8	95.0	0	22.5	1.3	10	22 Aug 2014
PIR	*Phytopthora infestans* resistance (leaf)	Resistant,susceptible	Greenhouse	Categorical Traits					Lenman et al. 2016	NA
LT	Leaf texture	soft/stiff	Field						10	26 July 2013
FT	Flowering time	early/late	Field						10	2013/2014

In order to relate phenotypes to each other, Spearman correlation was calculated based on trait values (Table 2). As could be expected, several growth phenotypes such as tuber yield (2013 and 2014; linear regression of R^2^ = 0.31 between seasons), height and growth rate are positively correlated with each other. These trait correlations are well-known in different potato growing systems (e.g. [12, 13]). The growth-related traits were negatively coupled to flowering time and senescence, which if late led to less tuber yield, reflecting the necessity for not too late development for high yielding cultivars in Nordic climates, which have relatively short growth seasons. Similarly, necrotic lesion development in field and HR-like response in the lab were negatively correlated with yield. The degree of HR-like lesions observed in the lab were clearly positively correlated with an increased number of necrotic spots observed in the field.

**Table 2:** Spearman correlation coefficients for the collected traits for progeny lines and parents

	Biotic Stress					Tuber						Leaf/shoot					
	DR	PIR	LAI	HRL	AIV	VT	TGF	YP13	YP14	TBS	NoT	H	SLS	GR	LT	NLL	FT
**DR**	1	*0.083*	– 0.18	– 0.099*	0.07*	0.11	0.29	0.037*	0.021*	– 0.067*	– 0.024*	0.26*	– 0.21*	0.26**	– 0.23*	– 0.28*	– 0.081*
**PIR**		1	– 0.12	0.16	0.28	– 0.15	– 0.019	0.091	0.094	– 0.41	– 0.076	0.14	– 0.098	0.12	0.05	0.038	– 0.036
**LAI**			1	– 0.29	0.18	0.11	– 0.071	0.025*	0.077*	0.18	0.034	– 0.048	– 0.32	– 0.022	0.1	– 0.041	– 0.12
**HRL**				1	– 0.25**	– 0.13	– 0.14	– 0.29**	– 0.53**	0.1*	0.11	– 0.41**	0.64**	– 0.4**	0.11	0.52**	0.4**
**AIV**					1	– 0.2	– 0.06	0.37**	0.52**	– 0.32*	– 0.081	0.32**	– 0.5**	0.36**	– 0.11	– 0.27**	– 0.31**
**VT**						1	– 0.041	0.059	0.046	– 0.22	0.31	– 0.0026	0.058	0.071	– 0.13	– 0.02	0.21
**TGF**							1	– 0.0059	– 0.06	– 0.04	– 0.39	0.25	0.16	0.27	– 0.096*	– 0.13	– 0.016
**YP13**								1	0.65**	0.013*	0.08	0.38**	– 0.47**	0.38**	– 0.21	**– 0.41**	**– 0.53**
**YP14**									1	– 0.017*	0.11	0.58**	– 0.68**	0.55**	0.008*	**– 0.49**	**– 0.62**
**TBS**										1	0.26	– 0.22*	0.22*	– 0.21*	– 0.037*	0.32*	0.15*
**NoT**											1	– 0.24	0.043	– 0.22	0.045	0.0018	– 0.055
**H**												1	– 0.7**	0.97*	– 0.15*	– 0.68**	– 0.5**
**SLS**													1	– 0.68**	0.011*	0.69**	0.63**
**GR**														1	– 0.23*	– 0.66**	−0.44**
**LT**															1	0.043*	– 0.012
**NLL**																1	0.64**
**FT**																	1

Significant correlations are indicated by ** *p* < 0.001 and *p < 0.05; highly significant correlations are indicated in bold and Italic. *DR* Dikeya resistance, *PIR P. infestans* resistance, *LAI* Lesions after Alternaria infection, *HRL* HR-like lesion, *AIV* Alternaria infection volume, *TGF* Tuber greening, field, *NoT* Number of tubers, *TBS* Tuber blight score, *YP14* Yield/plant 2014, *VT* Visible tubers/plant, *YP13* Yield/plant 2013, *SLS* Senescence late season, *GR* Growth rate, *H* Height, *NLL* Necrotic leaves, late-season, *LT* Leaf texture, *FT* Flowering time

Of the disease traits measured, Alternaria infection in tubers correlated positively with yield, growth rate and height, whereas less Alternaria infection was correlated with later senescence in the field, and late flowering time as reported earlier [14].

*P. infestans* susceptibility did not correlate with any of the other measured traits, and this reflect the 1:1 presence or absence of this single dominant resistance gene recently identified by us to drive the late blight resistance in this progeny population [10]. Interestingly, a number of transcripts changing in abundance were identified as possible effects of the presence of the R-gene, discussed below.

### Transcriptome assembly and annotation

In total, 875.6 million paired-end reads were sequenced from the 34 segregating potato lines. The average read count per line was approximately 25 million reads. After read quality control, only cleaned reads with a quality score higher than 20 and read length longer than 20 bp were considered for the analysis.

Sequence variations between genotypes can affect read alignment. Therefore, we generated a master transcriptome based on sequence data from all lines and the two parents to map all reads back to. In order to build a master transcriptome, all the sequenced samples from the 34 lines were pooled. The de novo assembly of the master transcriptome yielded a total of 212,536 contigs with a minimum length of 201 bp, a N50 value of 1177 bp and an average length of 724 bp. To ensure quality of the assembly, reads were mapped back to the master transcriptome and a satisfactory average of 83% successfully mapped reads per sample was achieved (Table S2. The assembly was further evaluated for sequence completeness through BUSCO, which identified 1258 out of 1440 ultra-conserved core proteins as ‘complete’ in the transcriptome assembly, corresponding to 87% complete genes. Sixty eight sequences were identified as ‘fragmented’ b BUSCO (Additional file 2: Table S3). This is slightly lower completeness than has been reported for potato transcriptomes, however it should be remembered that the master transciptome originates from leaf tissue only and excludes roots, possibly leading to a lower completeness. The master transcriptome was annotated against the ITAG and PGSC genome annotations and the Uniprot database by BLASTX. A total of 185,189 (87.13%) ITAG annotations, 178,635 (84.04%) PGSC annotations and 122,104 (57.45%) Uniprot annotations were associated to assembled transcripts. All the transcripts were matched against sequenced potato genome by BLASTN to identify genomic locations of assembled transcripts. A detailed summary of Uniprot, ITAG, PGSC and Gene Ontology (GO) annotation along with transcripts identifiers can be found in Additional file 2: Table S4.

### Transcript abundance and differential gene expression analysis

Transcriptome abundance estimation was performed to find common transcripts among all 34 progeny and two parent lines. Common transcripts were identified from normalized read count with FPKM value greater than or equal to 1 and it revealed that 21,708 (10.25%) transcripts were expressed in all 34 progeny lines and both parents, Desirée and SW93–1015. As expected, highly abundant transcripts were related to plant photosynthesis and central metabolism. A list of commonly expressed transcripts along with their functional annotation is given as Additional file 3: Table S5. five thousand four hundred eighty-four transcripts with annotations were differentially expressed between lines (FDR < 0.05, 2-fold change). A list of differentially expressed transcripts is given in Additional file 3: Table S6. A principle component analysis (PCA) of the transcriptome data was done, which shows a distinction between the parents as well as that several lines are different compared to the parents (Additional file 4: Fig. S1a), and numbers of differential gene expression in progeny lines in comparison to parents are shown in Fig. S1b together with the method to calculate these in Methods S1 (Additional file 4: Fig. S1b and Methods S1). GO analysis did not show clear overrepresentation of certain categories (data not shown).

### Gene expression analysis for categorical traits

Phenotype of three traits were categorical: *Phytophthora infestans* resistance (PIR; resistance or susceptible), Leaf texture (LT; soft or stiff), and Flowering time (FT; early or late). In addition to the correlation analysis, we therefore performed a differential gene expression analysis for categorical traits by DESeq2 (*p* < 0.05) using the trait categories as replicates. However, significantly differentially expressed genes were found only for *P. infestans* resistance. A total of 83 transcripts were differentially expressed between resistant and susceptible lines (p < 0.05; Fold Change> 2; Additional file 5: Table S7, see below**)**.

### Phenotype and transcriptome correlations to identify trait-associated transcripts

For transcriptome-to-phenotype association, we only selected transcripts with an expression count (FPKM) more than five in at least eight of the 34 progeny lines and the two parents. A total of 18,542 transcripts fell within these selection criteria. This stringent threshold led to a considerable loss of transcripts identified by Trinity (212,536). To test the effect of the high loss of transcripts we matched the Trinity-identified transcripts, 212,536 and 18,542, respectively, to the PGSC genome (BLAST, e-value<1e-05), and found that these represented 28,933 and 10,926 unique PGSC transcripts, respectively. The true number of transcripts present in the leaf tissue probably lies somewhere in between these two numbers, but we decided to maintain the threshold chosen in order to avoid false positives. Previously ca 22,000 transcripts were detected in the leaf transcriptome in Phureja [15].

**Fig. 1:**
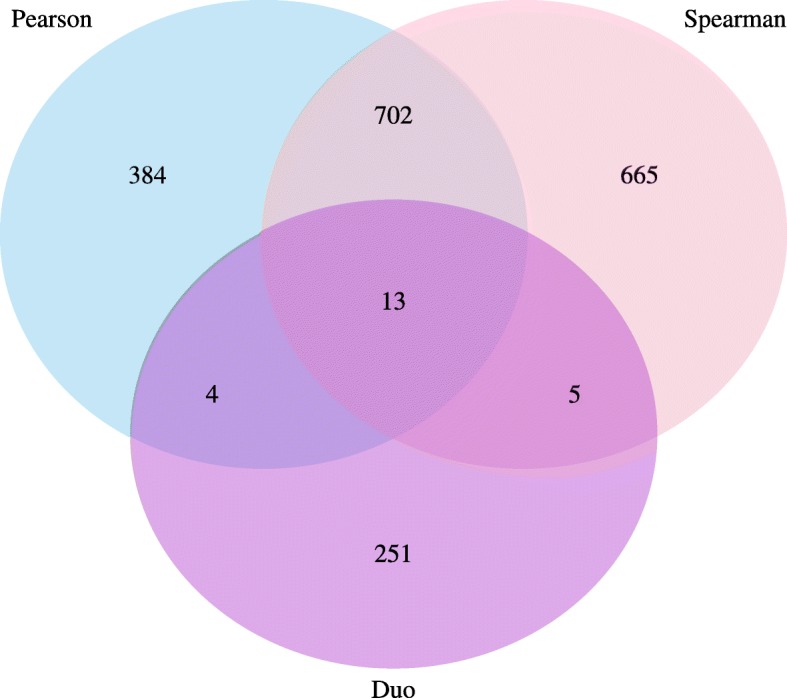
Venn diagram showing the number of transcripts shared between the Pearson, Spearman and Duo metrics at respective threshold (r = ≤ − 0.5 or ≥ 0.5, r = ≤ − 0.5 or ≥ 0.5 and ≥ 0.65)

In order to explore gene expression and phenotype relationships, expression data and the phenotypic data were combined and scaled through mean and standard deviation. Pearson correlation coefficient (PCC), Spearman correlation coefficient (SCC) coefficient and DUO metric were calculated between transcript expression and trait values across all 34 progeny lines and the two parents. Among 18,542 transcripts subject for transcriptome-to-phenotype association, we identified 1685, 2185 and 273 transcripts related to traits by Pearson (r = ≤ − 0.5 or ≥ 0.5), Spearman (r = ≤ − 0.5 or ≥ 0.5) and DUO (≥0.65), respectively. The number of transcripts identified from Pearson, Spearman and DUO, for each trait and common transcripts between methods, and among all three methods, are given in Table 3 and Fig. 1. The top 20 associated transcripts for each trait and PCC and SCC correlation are given in Additional file 6: Table S8 and S9.

**Table 3:** Number of correlated transcripts for the collected traits at thresholds Pearson (r = ≤ − 0.5 or ≥ 0.5), Spearman (r = ≤ − 0.5 or ≥ 0.5) and DUO (≥0.65), as well the overlap between the correlation metrics

	Traits	Abbr.	DUO	Pearson	Spearman	Duo∩ Pearson	Duo∩ Spearman	Pearson∩ Spearman	Pearson∩ Spearman∩ Duo
Biotic Stress	Dikeya resistance	DR	33	8	8	1	0	3	0
	*P. infestans* resistance	PIR	52	16	23	1	2	16	1
	Tuber blight score	TBS	0	19	23	0	0	4	0
	Lesions after Alternaria infection	LAI	0	224	244	0	0	142	0
	Alternaria infection volume	AIV	5	18	18	0	0	4	0
	HR-like lesion	HRL	4	56	65	0	1	38	0
Tuber	Tuber greening, field	TGF	0	20	2	0	0	1	0
	Number of tubers	NoT	0	464	790	0	0	384	0
	Yield/plant 2014	YP14	0	9	21	0	0	7	0
	Yield/plant 2013	YP13	25	57	11	0	0	4	0
	Visible tubers/plant	VT	0	41	7	0	0	0	0
Leaf/shoot/flower	Senescence late season	SLS	75	62	43	6	5	29	4
	Growth rate	GR	0	7	10	0	0	6	0
	Height	H	5	8	14	0	0	6	0
	Necrotic leaves, late-season	NLL	48	39	37	5	6	24	5
	Leaf texture	LT	26	11	14	4	4	9	3
	Flowering time	FT	0	26	33	0	0	23	0

### Differences between DUO and Pearson/Spearman

**Fig. 2:**
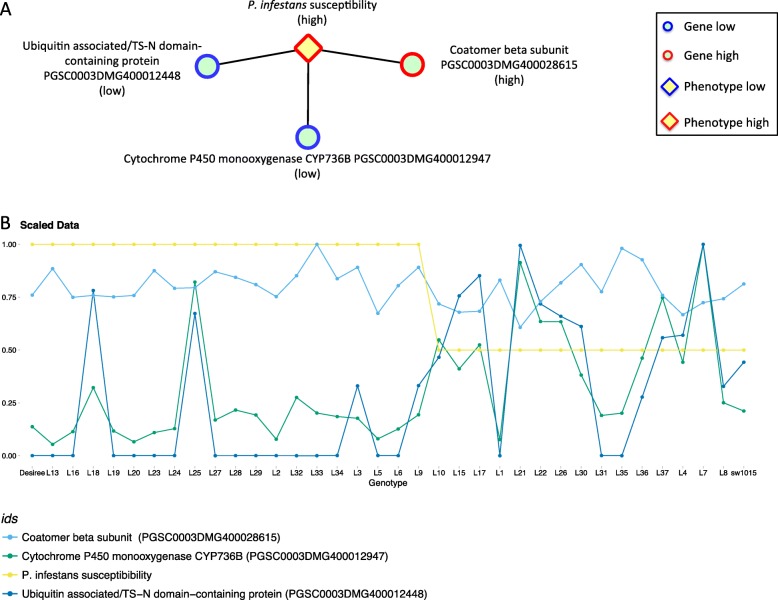
Three of the genes associated with the *P. infestans* leaf resistance (**a**) and their respective line plots over the progeny lines and parents (**b**)

To link transcripts to traits, first Pearson correlation and Spearman’s rank correlation coefficients were applied since they complement each other as the relationship between traits and transcripts can be expected to exhibit both linear and non-linear relationships. As can be seen in Table 3, most associated transcripts show a clear overlap between the methods. However, for some traits such as Tuber blight score there is a clear discrepancy. For a given transcript-trait pair, the DUO similarity metric measures the co-occurrence of extreme values between those two objects. DUO is unique in that it returns separate values for each type of correlation (e.g. high values of the first with low values of the second) thereby reducing errors induced by heterogeneity in the samples. Though the DUO metric finds lower numbers of associations than Pearson and Spearman, it does discover trait-transcript associations missed by those metrics (for example see Fig. 2) and can thus be seen as complementary in nature. In Additional File 7: Fig. S2 the correlation network (*p* ≥ 0.65) based on DUO shows that some transcripts correlate with more than one trait, such as late senescence (SNS) and few necrotic lesions in field (NLN). More tests with empirical data and biological validations of gene-to-trait relations are needed, however, to better benchmark the methods against each other.

### Identified trait-associated transcripts

#### Biotic factors

The population was tested with three different pathogens, which are common problems in European potato cultivation: *Phytophthora infestans* causing late blight, *Alternaria solani* causing early blight and *Dickeya solani* causing blackleg disease.

We identify several transcripts associated to the *P. infestans* leaf resistance (late blight) with DUO as well as Pearson and Spearman correlations (see below). Transcripts are regulated both positively and negatively with resistance. Interestingly, these can be seen as effects of the identified Rpi-ABPT (closest sequence identity with DMG400032576) gene in this population [10], and indicate that this receptor has some kind of basal role without challenge by *P. infestans* or other stresses as the transcriptomes were generated from plants in optimal conditions in controlled environments.

Low levels of the Rpi-ABPT gene transcripts were detected in some, but not all resistant lines, and this transcript did therefore not pass the threshold to be included in the association analyses (see Methods and Material; data not shown). The regulated genes also associates to the study of secreted peptides in this population, where all peptides used to predict resistance were less abundant in the resistant combination even without any disease [11]. This data also adds to the discussion about cost and trade-offs of resistance [16].

In the differential expression analysis by DESeq2 only up-regulated transcripts as a consequence of leaf blight resistance passed the threshold (adjusted *p* < 0.05; Fold Change> 2), in total 56 unique genes from different chromosomes (Additional file 5: Table S6**)**. As can be seen in Additional file 8: Fig. S3 there is a substantial number of transcripts only detected by differential expression analysis. It should be noted that in the differentially expression analysis all detected transcripts were included, and the majority of transcripts only identified by in this analysis had low expression levels and consequently did not pass the threshold to be included in the association analyses (see above and Materials and Methods). Among differentially expressed genes were several related to disease resistance, whereof three fall in the large TIR-NBS-LRR resistance gene family (DMG400007743, DMG400032576 and DMG400011529) and other into smaller families unique to potato according to PLAZA4.0, such as Late blight resistance protein (DMG400046318) and Plant disease resistant protein (DMG400044298). Yet another NBS-LRR family member annotated as NRC1 (DMG400007462) was detected by Pearson correlation as negatively correlated with *P. infestans* resistance. Other noteworthy transcripts only identified by differential expression analysis are a Rhicadhesin receptor (DMG400037420), an Erwinia induced protein 2 (DMG400023621) and several members of Zinc knuckle family proteins (DMG400036915, DMG400022758, DMG400028865). Not much is known about their role in resistance, but the Zinc knuckle family has previously been shown to be expanded in Solanum species with high level of *P. infestans* resistance [17].

Few transcription factors were associated to leaf blight resistance. However, a TCP transcription factor (DMG400016363) was less expressed in lines with leaf resistance towards *P. infestans*, indicating that this transcription factor can be a putative susceptibility factor. This is a diverse group of transcription factors, many of which are involved in organ development but also in plant defence and in effector-triggered immunity (ETI) [18]. In fact, in a protein-protein interaction network of effectors and Arabidopsis immune proteins, no less than three of the identified immune interactors belonged to the TCP family. These were found to be directly targeted by effectors from both bacterium *Pseudomonas syringae* and the obligate biotrophic oomycete *Hyaloperonospora arabidopsidis* (Hpa), and single mutants of all these three TCPs lead to disease susceptibility of two normally avirulent Hpa isolates [19]. Apart from the TCP transcription factor DUO picks up a negatively associated MADS transcription factor (DMG400000008) with weak similarity to MADS-box transcription factor 50 (OsMADS50) in rice.

Three of the most highly correlated transcripts with *P. infestans* leaf resistance were an Arabinogalactan protein (DMG402032565), a Sodium/potassium/calcium exchanger (DMG400006339) and an Associate of C-myc transcript (DMG400025428) with unknown function, all of which were detected by the differentially expression analysis as well. A transcript encoding a chromodomain remodeling complex (DMG400006925) was clearly positively regulated with resistance and highly similar to AtBAF60 (AT5G14170), which was recently shown to mediate repression of seedling growth [20].

Flavonoids are generally associated to plant defence and a flavonoid 3-hydroxylase (DMG400006354) was found to be negatively regulated with leaf resistance also by differential expression analysis. Furthermore, DUO identified a Leucoanthocyanidin dioxygenase (DMG400003091), which is similar to the AtSRG1 (SENESCENCE-RELATED GENE 1) involved in flavonoid biosynthesis.

In this progeny population, tuber blight resistance was not linked to foliar blight resistance [11, 21], and there were only two transcripts, which correlated with *P. infestans* resistance in both leaves and tubers. The two transcripts, actin-depolymerizing factor 2 (DMG400027752) and FGFR1 oncogene partner (DMG400032551), are involved in cellular organisation were positively correlated to resistance. The latter is weakly similar to Arabidopsis AtTON1A, which mutant exhibit abnormal cell growth and patterns of division in epidermal and cortical cells [22].

However, a weak correlation existed between Alternaria tuber and tuber blight resistance (Table 2) but only one transcript encoding a gene of unknown function with a Rhodanese-like domain (DMP400033223) was correlating with both traits. For tuber resistance three WRKY transcription factors (DMG400020608; DMG400009530; DMG400028520) and a VAMP protein SEC22 (DMG400016420) linked to the enriched GO terms biotic stimulus and chitin response, were all negatively correlated with tuber blight resistance (Additional file 6).

*D. solani* susceptibility seems to be a quantitative trait in potato and no single gene resistance has so far been identified, which makes this trait especially interesting to study by expression correlation. Still, DUO, which should efficiently identify binary differences, found a number of putative receptors and transcripts associated to signalling. For example a transcript (DMG401017405) highly expressed in resistant lines, and which has homology to a gene encoding a GPCR-type G protein in Arabidopsis (AT4G27630). This protein is membrane-bound and reported to bind the phytohormone ABA, but does not show transcriptional regulation by abiotic stress [23]. A receptor serine-threonine protein kinase (DMG400017596) highly homologous to CDG1 kinases was also highly expressed with increased resistance. Contrarily, a transcript with low expression in resistant lines was an Arabidopsis nitrate transporter homolog (DMG400013815). An Arabidopsis protein has been reported to act as a sentinel of nitrogen availability, and to be associated with increased susceptibility to infections by bacteria Pseudomonas [24]. Three other transcripts associated by DUO with low expression in Dickeya resistant lines were a disease resistance protein in the CC-NBS-LRR class (DMG400018462), a Cf-2.1-like receptor (DMG400006655) and a reticuline oxidases (DMG400021111). The latter is regularly appearing as part of induced resistance responses in potato [25]. Much fewer transcripts were associated to *D. solani* resistance by Pearson and Spearman, but a Permease I homolog (DMG400024588) overlapped between the methods and was more lowly expressed in more resistant lines.

Only a handful of transcripts were matched to Alternaria leaf infections with DUO. However, GO analysis of transcripts identified by Pearson correlations showed an enrichment of transcripts linked to Systemic acquired resistance as well as jasmonic and ethylene responses (Additional file 9). Among these was a Jar1 homolog (DMG400033879), for which lines with higher expression show increased resistance. Jar1 catalyzes the formation of jasmonyl-isoleucine (JA-Ile) conjugate, which in its turn promote the interaction between JAZ1 and COI1 in the jasmonate signalling pathway. Similarly, an EIN3 transcription factor (DMG400005915) was generally more lowly expressed in more resistant lines. EIN3 interacts with MYC2, MYC3 and MYC4 to regulate plant defense. This indicates that these hormone pathways are varying depending on resistance level even in the absence of the pathogen.

#### Development and yield traits

To investigate the development and yield-related traits, we related the subset of 84 transcripts associated by DUO to these traits to Arabidopsis homologous genes (Additional file 10: Table S10). Indeed, several of the Arabidopsis homologs are related to growth and development, and several families of transcription factors are represented, and this is also true for the GO analysis (Additional file 9). However, transcripts related to the yield data of 2013 and 2014 did not show enrichment of specific GO terms.

Photosystem II CP47 chlorophyll apoprotein (DMG400046303) positively correlated according to Pearson and Spearman with yield, as was the gene encoding a Photosystem Q(B) protein (DMG400004211), also active in the light reactions. Contrary, transcript levels of UBX-domain protein UBX2 (DMG400029482) and an Acyl-CoA synthetase (DMG400020593) were negatively correlated to yield in both years. In Arabidopsis, the knock-out of a member of the UBX family led to increased growth rates [26].

For the highly correlated traits height and growth rate, the GO term “regulation of timing of transition from vegetative to reproductive phase” was overrepresented (Additional file 9). Associated to this term was a MADS box transcription factor (DMG400022748) and Flowering locus T (DMG400016179), which were negatively and positively correlated to the traits, respectively. Highly negatively correlated with height and growth rate was a GLABRA2 expression modulator (DMG400023632), which homolog in Arabidopsis regulates cell division and is part of the ABA signalling pathway [27]. High expression of this gene was also linked to less necrotic leaves in the late season. Furthermore, a putative squamosa promoter binding protein (DMG400022824), which is a transcription factor in Arabidopsis involved in regulation of flowering [28], was highly negatively correlated with height and growth rate, but positively related to early senescence.

Number of tubers showed many highly positively and negatively correlated transcripts. There was a GO enrichment for sugar-meditated signalling pathway (Additional file 9). Examples were a positively correlating mannitol transporter (DMG400011964) and a hexose transporter (DMG400009994), highly similar to Arabidopsis TMT2 (AT1G45249). Negatively correlating was an ABRE binding factor (DMG400008011), which is a transcription factor involved in ABA signalling. Also related to ABA signalling was a NAC domain transcription factor (DMG400009245; AT1G01720).

The leaf texture trait were associated with several transporters, such as an ammonium transporter 1 (DMG400028710), and cell wall proteins such as a polygalacturonase (DMG400002931).

Late flowering was associated to the increased expression of a gene encoding a Tuftelin interacting protein, which is highly similar to AtNTR1 (AT1G17070). A mutation in this Arabidopsis gene alters the circadian period [29]. Associated with late senescence was also a zinc finger protein (DMG401017733), which homolog in Arabidopsis is suppressing late flowering time [30]. Early senescence was also associated to higher expression of a Serine/threonine-protein kinase PBS1 homolog (DMG400004594). PBS1 is well described in Arabidopsis and is involved in PAMP-triggered immunity (PTI) [31].

### Verification of marker transcripts by qPCR

**Fig. 3:**
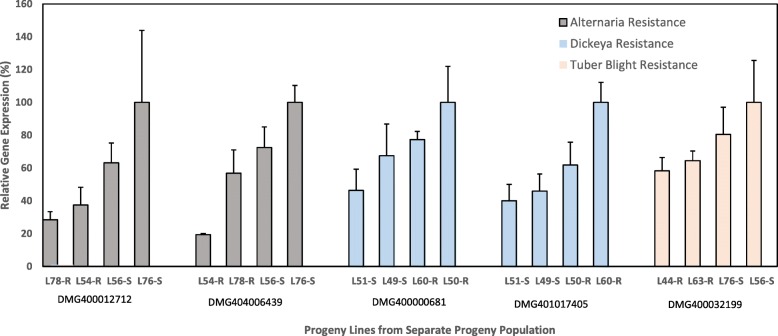
The expression of five putative transcript biomarkers as determined by qPCR. Error bars show technical variation as standard deviation

To verify some of our putative transcript biomarkers expression was analysed in a separate progeny population derived from an independent crossing using the same parents. Four lines with contrasting resistance and susceptibility for either early blight, Dickeya or tuber blight was chosen. The expression of five of these putative transcript biomarkers are shown in Fig. 3 and all showed a trend with differences in expression between the pairs of more resistant and more susceptible lines as could be expected.

Thus, at least when crossing the same parents these results confirmed Glycerol-3-phosphate dehydrogenase (DMG400012712) and Aldo/keto reductase (DMG404006439) as biomarkers identified by DUO for early blight. Furthermore, an AP2 domain class transcription factor (DMG400000681) and GPR89A (DMG401017405) could be confirmed for Dickeya resistance, the latter of which was identified by DUO. Finally, the expression of a peroxidase (DMG400032199) was as expected in the separate progeny population for tuber blight disease.

## Conclusions

We identify well-known as well as novel transcripts associated with 17 traits important for potato as a crop. This approach can be used to link the transcriptome and other molecular expression data to traits in future characterizations in segregating populations and elite material. In this study, we verify the expression pattern for five of the putative transcript markers in an independent progeny population However, further studies are needed to establish the predictive value of the transcript markers and profiles associated to the different traits in this study by testing other potato progeny populations, breeding lines and cultivars as well as over additional growing seasons and climates. We also show the benefit of analysing this type of data with different association methods. To this end, we try the novel method DUO on transcriptome data for the first time. DUO calculates the co-occurrence between phenotype value and expression level and are, thus, clearly different from the more regularly used Pearson and Spearman correlation.

## Materials and methods

### Plant growth, phenotyping, sample collection and preparation

The tetraploid parents, the cultivar Desirée obtained from Plant Science Sweden AB and the breeding line SW93–1015 obtained from Svalöf Weibull AB, have previously been described in Ali et al. 2012 and 2014 [21, 32]. The segregating population has also been described in relation to *P. infestans* leaf resistance screening data [10]. Field trials were conducted in Borgeby (55°45′5.8″N 13°2′13.5″E), Sweden and phenotypes were recorded in 2013 and 2014. Field growth conditions and managements have previously been described in Chawade et al. [11]. Table 1 contain information about number of plants or tubers that were investigated in each phenotype study, and under what conditions it was carried out. Dickeya scoring of in vitro plants was done according to [33]. *P. infestans* resistance on tubers (tuber blight) were assessed as described in [21], and the Phytophthora resistance data on leaves (late blight) is described in Lenman et al. 2016. Alternaria resistance in leaf and tuber was carried as in [34]. Nectrotic leaves late season and senescence were scored as a percentage of leaves affected by lesions and senescence, respectively, in the field. Degree of HR-like lesion was scored 1–6, with 1 denoting no necrotic leaves in the field.

For RNA extraction, potato plants were grown under controlled conditions (the Biotron at SLU Alnarp) with 16 h/8 h day and night regime with a fluorescent lamp light intensity of 200 μmol m-2 s-1. The temperature was set to 20 °C and the relative humidity was 65%. Pots were circulated every week to avoid positional effects. All sampling was done before flowering and and 10 fully expanded leaflets from the middle part of two plants were pooled and RNA extracted as described Ali et al. 2014 [32].

### Sequencing, de novo transcriptome assembly and annotation

Samples were sequenced through Illumina sequencing platform HiSeq 2000 as paired-end reads (2 × 100 bp) at BGI (Shenzhen, China). Sequences were deposited in ArrayExpress (E-MTAB-5996). Sequencing reads were trimmed by removing adapter sequences and low quality sequences with an average quality score of less than 20 were removed using Nesoni clip (v0.128). Reads with a length of less than 20 bp were also removed [35]. The remaining reads were assembled into a master transcriptome using Trinity version trinityrnaseq_r20140717 with default parameters [36]. To assess the quality of the transcriptome assembly, sequencing reads were mapped back to the assembled transcripts using the bowtie2 aligner [37]. The completeness of the transcriptome assembly was estimated using BUSCO [18], run on default parameters to evaluate the completeness of an assembly through estimating the presence and completeness of conserved genes. Assembled transcriptome data were annotated through BLASTX against the UniProt database. BLASTX was used for sequence similarity search against the NCBI non-redundant protein database using an e-value cut-off of 1e-10. Transcripts were also annotated against PGSC DM v 3.04 [38] and ITAG 2.3 annotation [39]. A GO enrichment analysis was done in GOEast using default settings [40], which includes a FDR adjustment (Yekutieli) and *p* < 0.1. GO terms assigned to transcripts using the best-performing functional annotation for the potato genome as determined by Amar et al. 2014 [41]. Sequence identities and gene families of identified transcripts were explored in PLAZA4.0 [42].

### Abundance estimation and differential gene expression analysis

RSEM (v1.2.7) was used for abundance estimation of the transcriptome assembly using the default parameters [43] to estimate the gene and isoform expression levels of RNA transcripts. The relative measure of transcript abundance was TPM (Transcripts Per Million) and FPKM (Fragments Per Kilobase of transcript per Million mapped reads). DESeq2 was used to find differentially expressed genes between the categorical traits [44, 45]. DESeq2 use negative binomial distribution method for differential expression analysis. DESeq2 were used in identification and analysis of differentially expressed genes and transcripts through Trinity version trinityrnaseq_r20140717 with default settings.

### Phenotype and transcript correlation

In order to establish phenotype and transcript relation, a core transcriptome was generated through filtering of transcripts by using FPKM values for crossing lines. Pearson correlation coefficient (PCC) and Spearman correlation coefficient (SCC) were calculated for each transcript and phenotypic traits for the lines in the crossing population and the parents [4]. We also explored the phenotype and transcriptome relationship through newly developed similarity measure, DUO (Discovery of synchronized gene expression modules using a vector-based correlation coefficient, Climer et al., in press). The gene expression data and trait data were combined into a matrix *M* in which each row represented a gene or a phenotype and each column represented a sample. A custom Perl script was used to scale each row by dividing each entry *x*_*i*_ by the maximum value of that row max(*X*), as in the following equation:

$$ {x}_i^{\ast }=\frac{x_i}{\max (X)} $$

where ${x}_i^{\ast }$ is scaled entry *i* of row vector *X*, *x*_*i*_ is the original entry *i* of row vector *X* and max(*X*) is the maximum value of row *X*. This ensures that all genes and phenotypes take values between 0 and 1.

The DUO similarity metric was then calculated between all pairs of genes and phenotypes according to the following procedure:

An upper threshold and a lower threshold were determined for the matrix *M*, such that 25% of the values in the matrix lay above the upper threshold, and labelled “high”, and 25% of the values in the matrix lay below the lower threshold, and labelled “low”. For each pair of features (rows in matrix) *A* and *B*, four comparisons were performed, namely the co-occurrence between high values in feature *A* and high values in feature *B*, low values in feature *A* and low values in feature *B*, high values in feature *A* and low values in feature *B*, and the co-occurrence between low values in feature *A* and high values in feature *B*, using to the following equation:

$$ {D}_{ij}=4{R}_{ij}\left(1-\frac{f_i}{1.5}\right)\left(1-\frac{f_j}{1.5}\right) $$

where *i* represents either high values of gene *A* or low values of gene *A*, *j* represents either high values of gene *B* or low values of gene *B, R*_*ij*_ represents the fraction of the vector length in which *i* and *j* co-occur and *f*_*i*_ and *f*_*j*_ represent the relative frequencies of *i* and *j* respectively.

### Phenotype-transcriptome correlation network

A transcript-trait association network was constructed across all the transcripts correlated with at least one trait, with transcripts and phenotypes represented as nodes and correlation values between transcripts and phenotypes represented as edge weights. For DUO network visualization, edges below 0.65 were discarded and the resulting trait-transcript associations were visualized in Cytoscape version 3.4.0 [46]. Each node represented high or low values of a gene or a trait, and edges represented the Duo similarity between those two nodes. Line plots were constructed using ggplot2 [41], RStudio [38], and various R packages/resources [37, 40, 42].

### Network comparison

The Pearson, Spearman and DUO networks were compared by counting the number of edges (transcript-trait associations) that were shared between these networks. This was done on a whole-network level (Fig. 2) and a per-trait level (Table 3). The Venn diagram was constructed using R [37], the R VennDiagram package [39] and RStudio [38].

### Verification by qPCR

The expression levels of five genes were validated by qPCR. Primers were designed with the help of Primer Blast (NCBI) according to the following criteria: predicted melting temperature of 59–71 °C, primer length of 18–24 nucleotides, product size of 100–250 base pairs (bp) and GC content of 40–60%. Primer sequences and efficiencies are given in Additional file 11: Table S14. For cDNA synthesis 500 ng of total RNA was transcribed to cDNA using SuperScript® III Reverse Transcriptase including degradation with RNase H according to the manufacturer’s protocol (InvitroGen). qPCR was performed with a CFX96 (ABI) using Power SYBR® Master Mix (InvitroGen) and PCR cycles ran according to the manufacturer’s recommendations. The comparative CT method was used for relative quantification of transcripts. The data was normalized to the 3 reference genes using the Pfaffl method [47]. The gene expression was calculated using Modified form of geNorm with multiple reference genes.

## Supplementary information

**Additional file 1.** Values including means of traits used for transcript association analysis (Table S1). NA denotes missing values.

**Additional file 2.** Evaluation of master transcriptome assembly by re-mapping sequencing reads by bowtie2 aligner (Table S2) and estimation of sequence completion by BUSCO (Table S3) as well as annotations by Uniprot, ITAG, PGSC and Gene Ontology (GO) of identified transcripts (Table S4).

**Additional file 3.** Expression values of transcripts in all progeny lines and the two parents (Table S5) and differentially expressed genes between progeny lines and parents (Table S6).

**Additional file 4.** Principle component analysis of transcriptomes of parents and lines (Fig. S1a). The number of differentially expressed transcripts of each line with respect to the parents Desirée and SW93–1015 (FDR < 0.05; 2-fold change, Fig. S1). A description on how gene expression of progeny lines with respect to their parental lines was determined is included in Methods S1.

**Additional file 5. **Differentially expressed genes comparing progeny lines resistant and susceptible to *P. infestans* (*p* < 0.05; Fold Change> 2; Table S7).

**Additional file 6.** Top 20 positively and negatively associated transcripts for each trait (Table S8) and transcripts with annotations and Pearson (PCC) and Spearman (SCC) correlations (Table S9).

**Additional file 7:** Figure S2. DUO gene and trait network at a 0.65 threshold as an interaction network produced in Cytoscape.

**Additional file 8: **Figure S3. Overlap between differentially expressed transcripts (p < 0.05; Fold Change> 2), Pearson and Spearman correlations, and DUO metric for the trait *P. infestans* resistance (PIR).

**Additional file 9.** Enriched gene ontology (GO) terms for transcripts associated by Pearson correlation to traits Tuber Blight Score (TBS), Alternaria infection volume (AIV), Number of Tubers (NoT), Height (H) and Growth Rate (GR).

**Additional file 10.** Potato transcripts associated to leaf and shoot traits related to 84 Arabidopsis homologs, including their functional evaluation, reports of phenotype and TAIR citations.

**Additional file 11.** Primer sequences for qPCR targets.

## Data Availability

RNA-seq data is deposited in ArrayExpress (E-MTAB-5996; https://www.ebi.ac.uk/arrayexpress/experiments/E-MTAB-5996/).

## Abbreviations

AIV: Alternaria infection volume in tubers
bp: Base Pair
CCC: Custom correlation coefficient
CT: Cycle threshold
DNA: Deoxyribo nucleic acid
DR: Dickeya response
FPKM: Fragments per kilobase of transcript per million
FT: Flowering time
GO: Gene ontology
GR: Growth rate
H: Plant height
HR: Hypersensitive response
HRL: Degree of HR-like lesions
ITAG: International Tomato Annotation Group
LAI: Lesions after Alternaria infection in leaves
LT: Leaf texture
NLL: Level of necrotic leaves in the late season
NLN: Necrotic lesions in field
NoT: Number of tubers
PAMP: Pathogen-associated molecular patterns
PCA: Principle component analysis
PCC: Pearson correlation coefficient
PGSC: The Potato Genome Sequencing Consortium
PIR: *P. infestans* resistance in leaves
PTI: PAMP-triggered immunity
qPCR: Quantitative PCR
R- gene: Resistance gene
RNA: Ribonucleic acid
RNA-seq: Ribonucleic acid sequencing
SCC: Spearman correlation coefficient
SLS: Senescence level in the late season
SNP: Single nucleotide polymorphism
SNS: Late senescence
SRM: Selective reaction monitoring
TBS: Tuber blight caused by *P. infestans*
TGF: Tuber greening in field
VT: Visible tubers close to soil surface
YP13: Yield per plant 2013
YP14: Yield per plant 2014
