# Supplementary material for: Linking crop traits to transcriptome differences in a progeny population of tetraploid potato
Source: BMC Plant Biol. 2020 Mar 18;20:120. doi: 10.1186/s12870-020-2305-x (PMC7079428; doi:10.1186/s12870-020-2305-x)
Supplement: Supplementary file 4 — Additional file 4. Principle component analysis of transcriptomes of parents and lines (Fig. S1a). The number of differentially expressed transcripts of each line with respect to the parents Desirée and SW93–1015 (FDR < 0.05; 2-fold change, Fig. S1). A description on how gene expression of progeny lines with respect to their parental lines was determined is included in Methods S1. [file 12870_2020_2305_MOESM4_ESM.pdf]

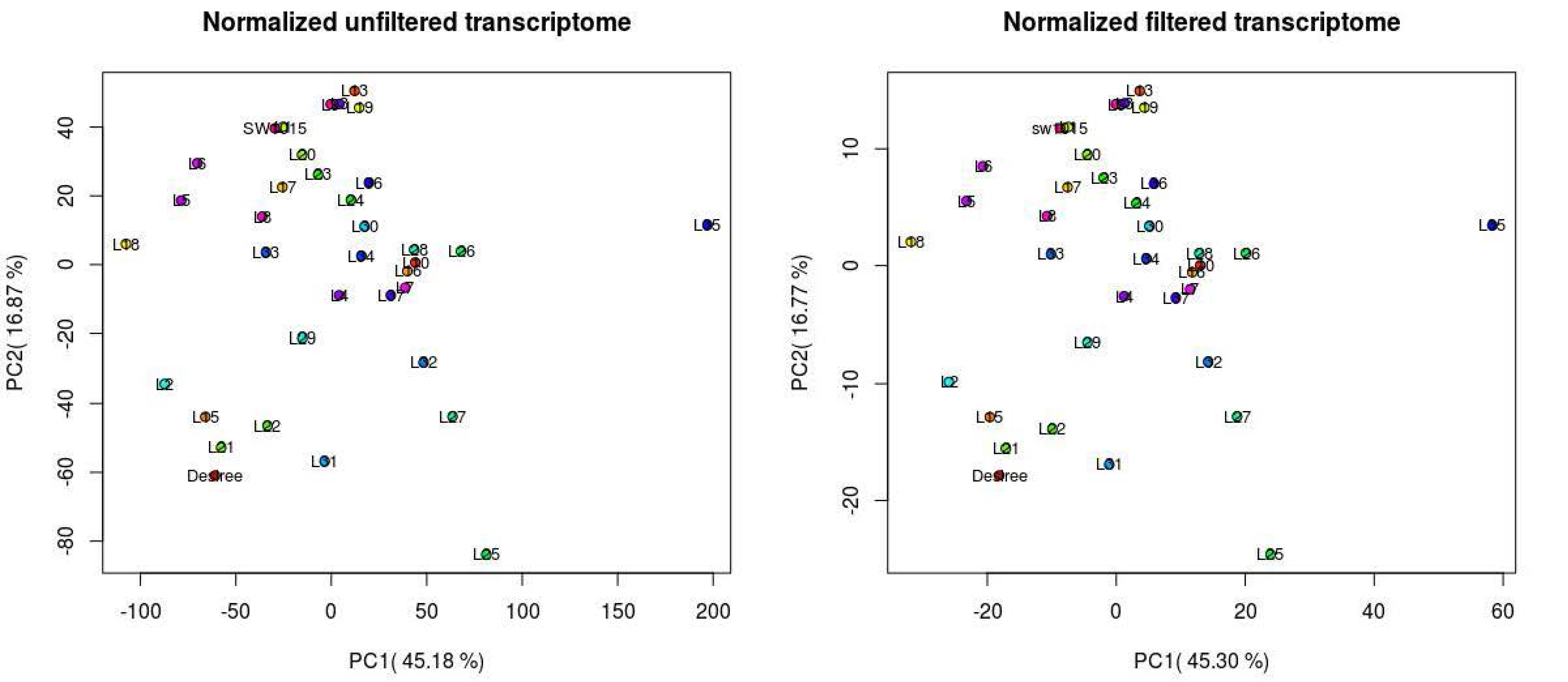
Normalized unfiltered transcriptome
PC1(45.18 %)
PC2(16.87 %)
Normalized filtered transcriptome
PC1(45.30 %)
PC2(16.77 %)

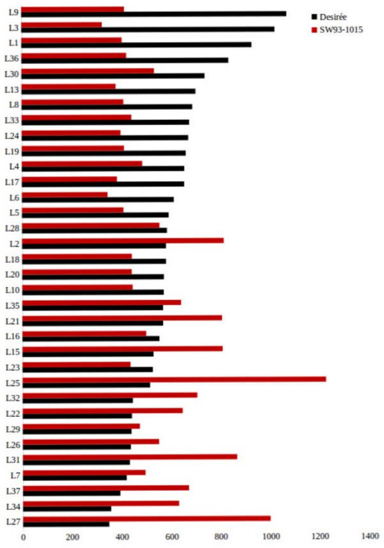
Desirée
SW93-1015
L9
L3
L1
L36
L30
L13
L8
L33
L24
L19
L4
L17
L6
L5
L28
L2
L18
L20
L10
L35
L21
L16
L15
L23
L25
L32
L22
L29
L26
L31
L7
L37
L34
L27
0
200
400
600
800
1000
1200
1400

Method S1: Gene expression of progeny lines with respect to their parental lines

A comparative study was performed to identify differentially expressed genes between progeny lines and the parents. Differential gene expression analysis was performed by edgeR [1] through Trinity version trinityrnaseq_r20140717to find differentially expressed genes between the individual progeny lines and the two parents separately.

Since no biological replicates existed to determine differentially expressed genes between the individual lines and the parents, we had to define a dispersion value for biological variation. This is possible in edgeR, and for biological variation in genetically identical organisms a dispersion value of 0.1 is suggested. We therefore tried dispersion values from 0.1 to 0.39 and we only had minor variation depending on the dispersion value in the number of differentially expressed genes with respect to the parents. So, we chose to go for the dispersion recommended for genetically identical organisms, 0.1.

1. Robinson MD, McCarthy DJ, Smyth GK: **edgeR: a Bioconductor package for differential expression analysis of digital gene expression data**. *Bioinformatics* 2010, **26**(1):139-140.
