# Supplementary figures and images for: Linking crop traits to transcriptome differences in a progeny population of tetraploid potato
Source: BMC Plant Biol. 2020 Mar 18;20:120. doi: 10.1186/s12870-020-2305-x (PMC7079428; doi:10.1186/s12870-020-2305-x)

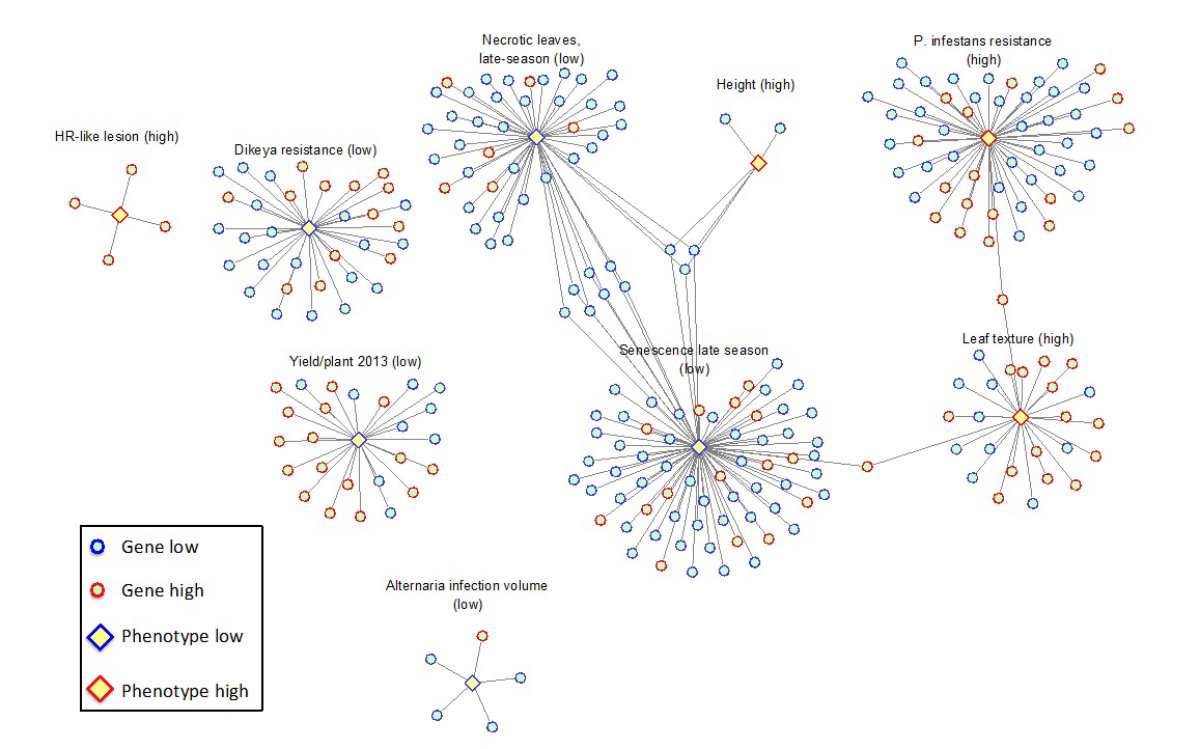

Supplement: Supplementary file 7 — Additional file 7: Figure S2. DUO gene and trait network at a 0.65 threshold as an interaction network produced in Cytoscape. [file 12870_2020_2305_MOESM7_ESM.jpg]

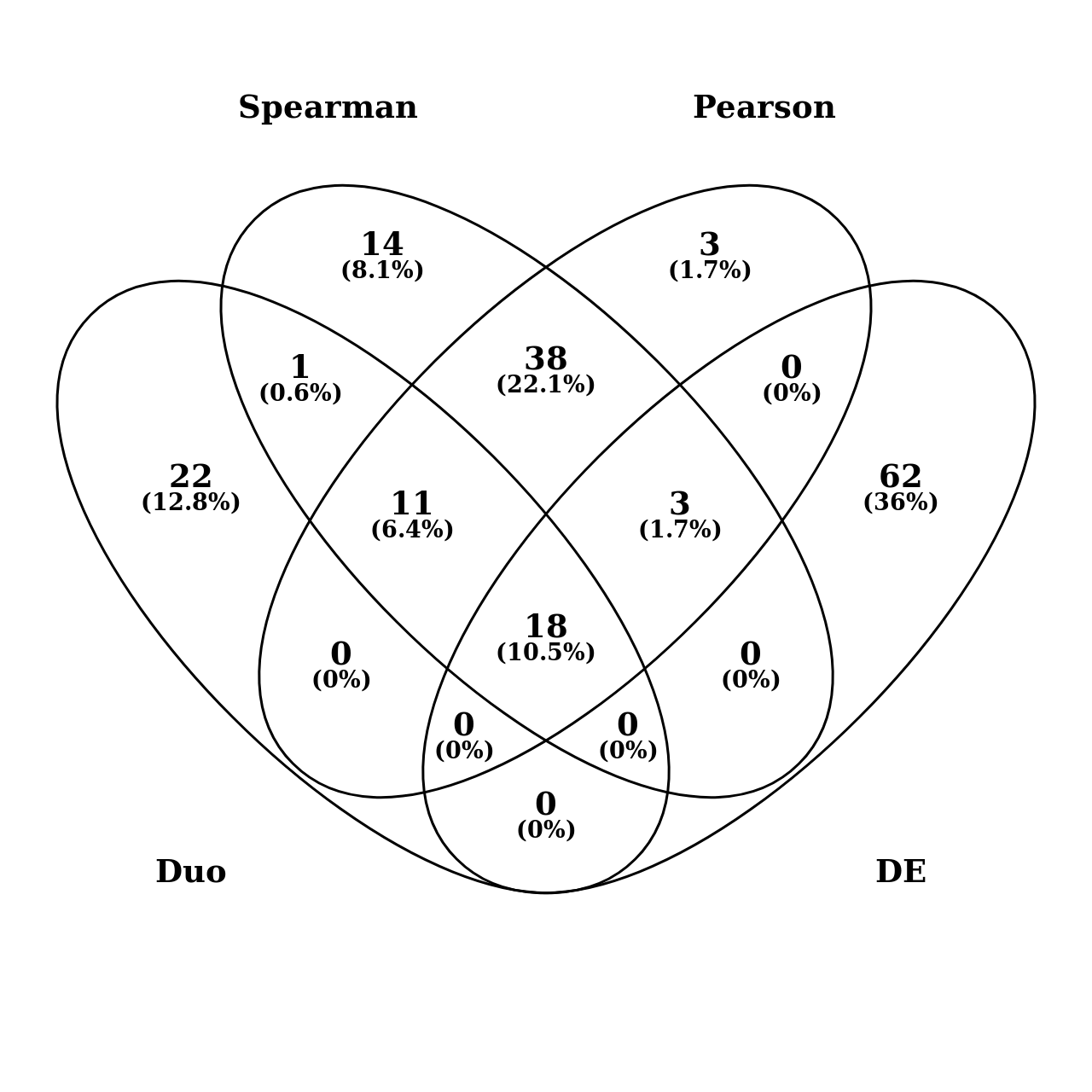

Supplement: Supplementary file 8 — Additional file 8: Figure S3. Overlap between differentially expressed transcripts (p < 0.05; Fold Change> 2), Pearson and Spearman correlations, and DUO metric for the trait P. infestans resistance (PIR). [file 12870_2020_2305_MOESM8_ESM.png]
