# Supplementary material for: Linking crop traits to transcriptome differences in a progeny population of tetraploid potato
Source: BMC Plant Biol. 2020 Mar 18;20:120. doi: 10.1186/s12870-020-2305-x (PMC7079428; doi:10.1186/s12870-020-2305-x)
Supplement: Supplementary file 10 — Additional file 10. Potato transcripts associated to leaf and shoot traits related to 84 Arabidopsis homologs, including their functional evaluation, reports of phenotype and TAIR citations. [file 12870_2020_2305_MOESM10_ESM.docx]

Table S9: 84 transcripts associated to leaf and shoot traits related to Arabidopsis homologs and their functional evaluation including reports of phenotype and TAIR citation

| **MapMan** | **Traits** | **Transcript ID** | **PGSC**  **Description** | **Arabidopsis**  **Orthologs** | **Arabidopsis Symbol** | **Arabidopsis**  **description** | **Phenotype** | **TAIR Reference** |
| --- | --- | --- | --- | --- | --- | --- | --- | --- |
| Hormone metabolism | Scenescence, late season | PGSC0003DMT400009831 | GID1-like gibberellin receptor | AT3G63010 | gid1b-1 gid1c-1 | alpha/beta-Hydrolases superfamily protein \| Chr3:23289717-23290998 | Only slight differences between mutant and wild type plants with regard to rosette radius and root length. | 17194763 |
| Hormone metabolism | Scenescence, late season | PGSC0003DMT400081370 | Lipoxygenase | AT3G22400 | SALK_044826 | PLAT/LH2 domain-containing lipoxygenase family | Developed more emergent (stage VIII) and lateral roots at 10 d. has a moderate increase in the length of the primary root. | 17369372 |
| Hormone metabolism | Necrotic leaves, late-season | PGSC0003DMT400084210 | Jasmonic acid-amino acid-conjugating enzyme | AT2G46370 | jar1-8 | Auxin-responsive GH3 family protein \| Chr2:19034579-19036369 | The jar1-8 mutant shows moderate insensitivity to MeJA in root growth inhibition assays. | 12084835, 18247047 |
| RNA  regulation | Scenescence, late season | PGSC0003DMT400072371 |  | AT2G38440 | scar2-3 | SCAR homolog 2 \| Chr2:16095550-16100851 FORWARD | Weak distorted phenotype reminiscent of arp2-arp3 complex mutant trichomes: branches of mutant trichomes are often twisted and variable in length. | 17267444 |
| RNA.  Regulation | Scenescence, late season | PGSC0003DMT400045726 | Zinc finger protein | AT1G30970 | suf4-1 | zinc finger (C2H2 type) family protein \| Chr1:11040613-11043593 | Suppress the FRI late flowering phenotype. Reduced H3K4 trimethylation at FLC locus. | 17079264 |
| RNA.  regulation | Necrotic leaves, late-season | PGSC0003DMT400036907 | Short-chain dehydrogenase/reductase | AT5G07350 | tsn1 tsn2 | TUDOR-SN protein 1 \| Chr5:2320344-2324892 REVERSE | Double mutant displays only mild growth phenotypes under nonstress conditions, but germination, growth, and survival are severely affected under high salinity stress. | 20484005 |
| Metal handling.  binding, chelation and storage | Necrotic leaves, late-season | PGSC0003DMT400069736 | Ferritin-2, chloroplastic | AT2G40300 | fer1-3-4 | ferritin 4 \| Chr2:16831501-16833214 REVERSE LENGTH=259 | Decreased leaf growth and CO2 fixation; impaired flower development and iron transport in the floral stalk. | 18826427 |
| Cell wall | Scenescence, late season | PGSC0003DMT400040409 | GDP-mannose pyrophosphorylase | AT2G39770 | vtc1 | Glucose-1-phosphate adenylyltransferase family | mutation enhances pathogen resistance | 15064386 |
| Transport | Scenescence, late season | PGSC0003DMT400041073 | Amt2 | AT2G38290 | amt2;1-1 | ammonium transporter 2 \| Chr2:16039672-16042291 | This mutant was used to construct a quadruple mutant: amt1;1-1, amt1;2-1, amt1;3-1, amt2;1-1. When this qko (quadruple knockout) was compared with a triple knockout with WT AMT2;1, it provided little evidence for a role of AMT2;1 in ammonium uptake in Arabidopsis roots. | 17693533 |
| Transport | Leaf texture | PGSC0003DMT400073887 | Ammonium transporter 1 member 2 | AT1G64780 | amt1;2-2 | ammonium transporter 1%3B2 \| Chr1:24061021-24062565 | The amt1;2-2 mutant appears to grow normally on media with different levels of ammonium or ammonium nitrate, but, seems slightly more resistant to methylammonium that wild type seedlings. High affinity ammonium uptake is reduced in the roots of amt1;2-2 mutants. There is no change in the level of AMT1;1, AMT1;3, or AMT2;1 transcripts for these mutants relative to wild type in low or high nitrogen conditions. | 17693533 |
| RNA.processing.ribonucleases | Leaf texture | PGSC0003DMT400023379 | RNA binding protein | AT3G62300 | SALK_059387 | agenet domain protein (DUF) | Homozygotes have a WT phenotype | 19795213 |

Table S9b: Identified genes related to biotic stress category

| **MapMan** | **Traits** | **Transcript ID** | **PGSC**  **Description** | **Arabidopsis**  **Orthologs** | **Arabidopsis Symbol** | **Arabidopsis**  **description** | **Phenotype** | **Reference** |
| --- | --- | --- | --- | --- | --- | --- | --- | --- |
| Hormone metabolism | P. infestans resistance | PGSC0003DMT400048153 | Aldehyde oxidase | AT2G27150 | aao3-4 | abscisic aldehyde oxidase 3 \| Chr2:11602551-11606176 | In the mutant, the ABA levels are reduced to around 50-60% of those of wild type. | 15574845 |
| Signalling.calcium | P. infestans resistance | PGSC0003DMT400059972 | Calreticulin | AT1G08450 | SALK_051336C | calreticulin 3 \| Chr1:2668008-2671800 REVERSE LENGTH=424 | Compromised in EFR but not FLS2 signalling. | 19717464 |
| Redox.thioredoxin | P. infestans resistance | PGSC0003DMT400007185 | Disulfide-isomerase | AT2G47470 | SALK_046705 | Encodes a protein disulfide isomerase-like \| Chr2:19481503-19483683 | Reduced seed set. Delayed embryo sac development results in defects in pollen tube guidance. | 19050167 |
| Cell  organisation | P. infestans resistance | PGSC0003DMT400056647 | Myosin XI-2 | AT5G43900 | xi-k / xi-2 | myosin 2 \| Chr5:17657241-17667413 REVERSE LENGTH=1565 | The overall growth of the xi-k / xi-2 double mutant is normal. In the leaf epidermis, Golgi stacks, peroxisomes, and mitochondria move more slowly in these mutant cells than in wild-type cells. Root hair length is reduced to ~20% of the wild type root hair length in these double mutants, but their root hair density is very similar to the wild type density. | 19060218 |
| Transport  metal | P. infestans resistance | PGSC0003DMT400064232 | Heavy metal cation transport atpase | AT4G37270 | hma1-ACT7 | heavy metal atpase 1 \| Chr4:17541987-17546352 REVERSE | In chloroplasts from hma1 mutant lines, Cu content was halved with respect to chloroplasts from WT. Under high light, hma1 mutants exhibited a strong photosensitivity phenotype leading to white leaves with restricted green regions. | 16282320 |
| Not assigned | P. infestans resistance | PGSC0003DMT400053253 | Binding protein | AT1G18260 | SALK_109430C | HCP-like superfamily protein \| Chr1:6279047-6282008 | Endoplasmic reticulum (ER)-associated degradation process is blocked and the unfolded protein response is activated in ebs5-5 mutant. | 21187394 |
| Stress  abiotic.heat | Dickyea Resistance | PGSC0003DMT400036235 | Heat shock protein binding protein | AT2G26890 | kam2-6 | DNAJ heat shock N-terminal domain-containing protein | deformed endosomes and protein storage vacuoles; aggregated endomembrane; swelled and electron-dense extracellular space; defects of growth axis of embryo | 17259264 |
| RNA  ProcessingRibonucleases | Dickyea Resistance | PGSC0003DMT400023379 | RNA binding protein | AT3G62300 | SALK_059387 | agenet domain protein (DOMAIN OF UNKNOWN FUNCTION | Homozygotes have a WT phenotype | 19795213 |
| Not assigned | Dickyea Resistance | PGSC0003DMT400044844 | Protein GPR89A | AT4G27630 | gtg2-1 | GPCR-type G protein 2 \| Chr4:13791980-13797033 | gtg2-1 mutant plants do not show any obvious phenotypic abnormalities and are indistinguishable from wild-type plants. | 19135895 |
